# Kinetics of neutralizing antibodies in patients naturally infected by H5N1 virus

**Published:** 2011-01-10

**Authors:** Philippe Buchy, Sirenda Vong, Simon Chu, Jean-Michel Garcia, Tran Tinh Hien, Vo Minh Hien, Mey Channa, Do Quang Ha, Nguyen Van Vinh Chau, Cameron Simmons, Jeremy J Farrar, JS Malik Peiris, Menno D de Jong

**Affiliations:** 1 Institut Pasteur in Cambodia, Phnom Penh, Cambodia; 2 HKU-Pasteur Research Centre, Hong Kong SAR; 3 Hospital for Tropical Diseases, Ho Chi Minh City, Vietnam; 4 Oxford University Clinical Research Unit, Ho Chi Minh City, Vietnam; 5 Department of Microbiology, University of Hong Kong, Hong Kong SAR China; 6 Department of Medical Microbiology, Academic Medical Center, University of Amsterdam, The Netherlands

## Background

Little is known about the kinetics of anti-H5 neutralizing antibodies in naturally H5N1-infected patients with severe clinical illness or asymptomatic infection. These data are essential for the design and interpretation of sero-epidemiological studies which are crucial to monitor the extent of asymptomatic or clinically mild H5N1 illness among humans or to identify risk factors associated with human contamination.

## Methods

Using H5N1 microneutralisation (MN) and H5-pseudotype particle-based microneutralisation assays (H5pp) we analyzed sera sequentially obtained from 11 severely ill patients diagnosed by RT-PCR (follow-up range 1 – 139 weeks of disease onset) and 31 asymptomatically infected individuals detected in a sero-epidemiological study after exposure to H5N1 virus (follow-up range: 1-2 month – 11 months after exposure).

## Results

Antibody kinetics measured by H5pp were similar to that from MN assay with a good correlation between the titers measured by the two methods (Spearman’s correlation coefficient of 0.79, p<0.001). Of 44 sera from 11 patients with H5N1 disease, 70% tested positive by MN (antibody titre ≥80) after 2 weeks and 100% were positive by 3 weeks after disease onset. The geometric mean MN titers in severely ill patients were 540 at 1-2 months and 173 at 10-12 months and thus were higher than the titers from asymptomatic individuals (149 at 1-2 months, 62.2 at 10-12 months). Fractional polynomial regression analysis demonstrated that in all severely ill patients, positive titers persisted beyond 2 years of disease onset, while 10 of 23 sera collected 10-11 months after exposure in asymptomatically infected individuals tested negative.

## Conclusion

We demonstrated a good correlation between the 2 tests used, hence confirming the validity of the H5pp test as a screening test in sero-epidemiological studies of H5N1 infection. Our data provide important novel insights into the dynamics of serological responses in patients with the full spectrum of clinical disease from severe though mild to asymptomatic H5N1 infection. Indeed, our results indicate that people with asymptomatic H5N1 infection have lower H5N1 antibody titers compared to those with severe illness and that in many asymptomatically infected patients the antibody titer decreased to levels below the threshold of positivity within one year. Hence delayed community sero-prevalence studies for H5N1 may underestimate the true burden of human infection.

Supported by the French Development Agency through the Surveillance and Investigation of Epidemic Situations in Southeast Asia (SISEA) project.

